# Metabolomics in Otorhinolaryngology

**DOI:** 10.3389/fmolb.2022.934311

**Published:** 2022-09-08

**Authors:** Antonio Noto, Cristina Piras, Luigi Atzori, Michele Mussap, Andrea Albera, Roberto Albera, Augusto Pietro Casani, Silvia Capobianco, Vassilios Fanos

**Affiliations:** ^1^ Department of Biomedical Sciences, University of Cagliari, Cagliari, Italy; ^2^ Department of Surgical Sciences, University of Cagliari, Cagliari, Italy; ^3^ Department of Surgical Sciences, University of Turin, Turin, Italy; ^4^ Department of Medical and Surgical Pathology, Otorhinolaryngology Section, Pisa University Hospital, Pisa, Italy

**Keywords:** Otorhinolaryngology, metabolomics, metabolites, precision medicine, sleep apnea, vertigo, hearing loss

## Abstract

Otorhinolaryngology (Ear, Nose and Throat-ENT) focuses on inflammatory, immunological, infectious, and neoplastic disorders of the head and neck and on their medical and surgical therapy. The fields of interest of this discipline are the ear, the nose and its paranasal sinuses, the oral cavity, the pharynx, the larynx, and the neck. Besides surgery, there are many other diagnostic aspects of ENT such as audiology and Vestibology, laryngology, phoniatrics, and rhinology. A new advanced technology, named metabolomics, is significantly impacting the field of ENT. All the “omics” sciences, such as genomics, transcriptomics, and proteomics, converge at the level of metabolomics, which is considered the integration of all “omics.” Its application will change the way several of ENT disorders are diagnosed and treated. This review highlights the power of metabolomics, including its pitfalls and promise, and several of its most relevant applications in ENT to provide a basic understanding of the metabolites associated with these districts. In particular, the attention has been focused on different heterogeneous diseases, from head and neck cancer to allergic rhinitis, hearing loss, obstructive sleep apnea, noise trauma, sinusitis, and Meniere’s disease. In conclusion, metabolomics study indicates a “*fil rouge*” that links these pathologies to improve three aspects of patient care: diagnostics, prognostics, and therapeutics, which in one word is defined as precision medicine.

## Introduction

Otorhinolaryngology deals with pathologies concerning ear, nose, larynx, cervical-facial area and the skull base in general. The term derives directly from the Greek “*ὠτορινολαρυγγολογία*” (otorinolaryngología), which stands for the study of the ear, nose, and larynx, respectively.

The field had a significant growth when an excellent anatomist, Antonio Maria Valsalva (1,666–1723), the favorite trainee of Marcello Malpighi, conceived the anatomical-clinical method for studying the human ear. Valsalva worked for over 16 years dissecting more than 1,000 animals, from which he comprehended the ENT anatomy (Fughelli et al., 2019). Valsalva made a detailed description of the cochlea, of the semi-circular canals and for the first time in history he described the presence of a limpid lymph within the inner ear and, as a result, he discovered the mechanisms that regulate acoustic perception. Moreover, his scientific rigor has influenced the way diagnoses are assessed nowadays, by looking at signs and symptoms, including palpation of the neck, inspection of the oral cavity and pharynx, assessment of the tympanic membrane and the external ear canal, and exploration of the internal cavities of the nose (Jellinek, 2006). However, even 229 years after the death of Valsalva, the molecular patho-physiological mechanisms of the ear, nose and throat have not been completely understood. The possibility to perform a biopsy and in-depth histological analyses, as for the cochlea tissue, is impossible without causing severe damage to the organ, thus, histopathology correlated to inner disease is only possible after death.

A new advanced technology, namely metabolomics, aims to understand the molecular phenotype of a particular disease. This approach generates a plethora of data, which can help to understand the mechanisms underlying biological processes and molecular functions (Mussap et al., 2021). Metabolomics is considered the endpoint of the “-omics” cascade, defined as the comprehensive measurement of low-molecular-weight metabolites in a biological specimen. Metabolites are either metabolic intermediates or metabolic end-products resulting from cellular metabolism. In the human body, they can result from human or microbial metabolism, environment, diet, and interactions among these factors (Nicholson et al., 1999; Mussap et al., 2016). To date, metabolomics is considered a research tool that can give novel insights in the diagnosis, treatment, and prognosis of ENT diseases (Mussap et al., 2016).

In this review a typical workflow for metabolomics study is discussed with a particular focus on ENT diseases such as head and neck cancer, allergic rhinitis, hearing loss, obstructive sleep apnea, noise trauma, sinusitis, and Meniere’s disease.

## General principles and typical workflow of metabolomics

The metabolome is highly dynamic and sensitive to perturbations, and for this reason there has been increasing need for quality improvement in the route from the laboratory analysis to the patient’s bedside. Generally, each metabolomic study includes three different phases named preanalytical, analytical and post-analytical (Wang et al., 2010).

The first and most crucial phase is the preanalytical phase which includes sample collection, transport, storage, and sample processing. Concerning sample collection and storage most of metabolomics studies use blood sample (either serum and plasma), saliva, and urine (Wishart, 2005).

Some of the ENT-related studies also use perilymph fluid which derives mainly from the cochlear blood flow. However, for blood sample there is still no general consensus on which anticoagulant should be recommended while, concerning saliva, urine and perilymph samples, some of the following rules are often not mentioned, such as: samples should be collected in a sterile bag to avoid metabolic modifications due to the bacteria metabolism; they should be centrifuged with high speed to avoid the action of active enzymes and the presence of cellular debris; they should be frozen at −80°C until analysis, and, lastly, they should be gently thawed on ice to avoid abrupt temperature variations. The analytical phase comprises the application of several techniques such as nuclear magnetic resonance spectroscopy (NMR) and gas or liquid chromatography coupled with mass spectrometry (MS). On one hand, MS approaches, despite destroying samples, are considered the gold standard in recognizing and quantifying metabolites because of their sensitivity in the picomolar and nanomolar ranges, but on the other hand, NMR can analyze the structure, and concentration of molecules without destroying the specimens (Go et al., 2015). Both techniques are thus considered complementary for metabolomics studies.

Moreover, the use of external standards, internal standards, or a combination of both is important to monitor the quality of the results (Liu et al., 2020).

As external standard the pooled quality control is of great utility: it consists of a pooled sample of all individual samples measured during a study and can be used to assess the repeatability and intermediate precision of all detectable metabolites present in the sample. As internal standard, the addition of labelled metabolites is also essential to monitor and correct, if necessary, metabolic responses in metabolomics studies. Each study should report data on quality control, intended at least as the ability of the system to generate reproducible results. Potential sources of errors in metabolites quantification by MS techniques are irreproducible extraction, derivatization, and degradation of derivatized metabolites. On the other hand, NMR spectroscopy may be difficult to interpret because of the presence of interfering compounds generating overlapping peaks. A further criticism may be the presence of uncharacterized metabolites, leading to an incomplete evaluation of the metabolome. Thus, it is recommendable to report in each study the analytical approach, the number of metabolites detected and those certainly identified with their concentrations; in addition, the number of unknown metabolites should be reported (Phinney et al., 2013).

The post analytical phase includes data analysis and metabolites annotation. The former is performed with two different approaches, univariate, and multivariate analysis (Wishart et al., 2018).

Univariate methods allow to reduce a great number of measured metabolites to only those showing the strongest response under the investigated conditions. However, univariate statistical approach fails to discriminate between groups if there are only minor differences on single molecule level. Multivariate analysis is appropriate for investigating the covariances and the correlations revealing the level of associations among the variables. Results obtained by multivariate methods should be confirmed by using a second set of “blind” samples. Moreover, further methods for confirming results are the calculation of several parameters: R2, estimating goodness of fit, and Q2, estimating goodness of prediction, the DModX test, identifying moderate outliers, and Hotelling T square test, identifying strong outliers. The latter step is crucial, and currently software solutions and extensive compound libraries facilitate the annotation process. However, the development of comparable tools for data annotation is essential, because the number of contaminants, artifacts, and misinterpretations support the uncertainty of the data (Wishart et al., 2018).

## Application of metabolomics in ear, nose and throat

In recent years, metabolomics in ENT has received growing attention and its application has contributed to understanding the metabolic mechanisms of initiation and progression of some ENT diseases, by identifying metabolic patterns shared among similar ENT conditions or rather characteristic of specific ENT diseases (Table 1).

**TABLE 1:** Selected characteristics of reviewed studies on ORL.

Author	Years	Sample size	Biofluid	Technique	Biomarkers Candidates	Results	Comments
						Increase (↑) or decrease (↓) biomarker concentration compared to the control group	
Head and neck cancers							
Almadori et al.	2007	127 subjects: 50 patients with oral and pharyngeal squamous cell carcinomas and 77 healthy controls	Saliva	HPLC-MS	Glutathione	Glutathione ↑	
Sugimoto et al.	2010	158 subjects: 69 oral cancer patients and 89 healthy controls	Saliva	CE-TOF-MS	Betaine; Isoleucine; Leucine; Phenylalanine; Tyrosine; Valine; Choline; Carnitine; Glycerophosphocholine; Cadaverine; Putrescine; Hypoxanthine; Ethanolamine; Trimethylamine; Tryptophan; Valine; Glutamic acid; Glutamine; Aspartate; Taurine; Lysine; Pyrroline hydroxycarboxylic acid; Pipecolic acid; Piperidine; Serine; β-alanine; Histidine	Isoleucine ↑	
						Leucine ↑ Taurine ↑	
						Valine ↑ Tryptophan ↑	
						Putrescine ↑ Alanine ↑	
						Pipecolic acid ↑	
						Choline ↑	
						Glycerophosphocholine ↑	
						Histidine ↑ Carnitine ↑	
						Threonine ↑ Glutamate ↑	
Tiziani et al.	2009	15 subjects with oral cancer and 10 controls	Serum	^1^H-NMR	Valine, Ethanol, Lactate, Alanine, Acetate, Citrate, Phenylalanine, Tyrosine, Methanol, Formaldehyde; Formic acid; Glucose; Pyruvate; Acetone, Acetoacetate; 3-hydroxybutyrate; 2-hydroxybutyrate; Choline; Betaine; Dimethylglycine; Sarcosine; Asparagine; Ornithine	Valine ↓ Ethanol ↓	The metabolomics profile of patients with oral cancer at the different stages of the disease was also evaluated
						Lactate ↓ Alanine ↓ Acetate ↓ Citrate ↓ Phenylalanine ↓	
						Tyrosine ↓ Methanol ↓ Formaldehyde ↓ Formic acid ↓ Glucose ↑	
						Pyruvate ↑ Acetone ↑ Acetoacetate ↑ 3-hydroxybutyrate ↑	
						2-hydroxybutyrate↑ Choline ↑ Betaine ↑ Dimethylglycine ↑ Sarcosine ↑ Asparagine ↑ Ornithine ↑	
Yonezawa et al.	2013		Serum and Tissue	GC-MS	Glucose; Ribulose; Methionine; Ketoisoleucine; Histidine, Aspartic acid, Glutamic acid, Asparagine, Lysine, Serine, Methionine; Alanine	In serum:	
						Glucose ↑ Ribulose ↑	
						Methionine ↓ Ketoisoleucine ↓	
						In tissue:	
						Histidine ↑	
						Aspartic acid ↑	
						Glutamic acid ↑ Asparagine ↑ Lysine ↑ Serine ↑ Methionine ↑ Alanine ↑ Glucose ↓	
Allergic rinitis							
Ma et al.	2020	28 allergic rhinitis patients and 28 healthy controls	Serum	UPLC-QTOF-MS	Bilirubin; Hypoxanthine; 15(S)-HETE*; Hexadecenoic acid; Urate; **13(S)-HPODE; Leukotriene D4; Stercobilinogen; N-succinyl-diaminopimelic acid; Chlorophyll b	Bilirubin ↑	*15(S)-hydroxyeicosatetraenoic acid; **13(S)-hydroperoxyoctadecadienoic acid
						Hypoxanthine ↑	
						15(S)-HETE ↑ Hexadecenoic acid ↑	
						Urate ↑	The metabolites were mainly involved in pathways of porphyrin and chlorophyll metabolism, arachidonic acid metabolism, and purine metabolism
						Leukotriene D4 ↓ Stercobilinogen ↓	
						N-succinyl-diaminopimelic acid ↓ Chlorophyll b ↓	
Zheng et al.	2021	73 patients with allergic rhinitis: 35 and 38 patients with single or double-mite subcutaneous immunotherapy respectively.	Serum	UPLC-QTOF-MS	*HETE; **HEPE;	In SM-SCIT and DM-SCIT patients:	*15(S)- hydroxyeicosatetraenoic acid
					***HODE;	*HETE ↓ **HEPE ↓	**5(S)-HETE, 12(S)- hydroxyeicosapentaenoic acid
					11(S)-HETE; 8(S)-HETE; 5(S)-HEPE; Arachidonic acid; Eicosapentaenoic acid (EPA); 9(S)- hydroperoxylinoleic acid (HPODE)	***HODE ↓	***13- hydroxyoctadecadienoicacid
						In DM-SCIT patients:	
						11(S)-HETE ↓	
						8(S)-HETE ↓ 5(S)-HEPE↓ Arachidonic acid↓ Eicosapentaenoic acid (EPA) ↓	
						In SM-SCIT patients:	
						HPODE ↑	
Yuan et al.	2022	28 patients with allergic rhinitis	Serum	UPLC-QTOF-MS	26 altered metabolites between:	Prostaglandin E2 ↑ Prostaglandin H2 ↑ Prostaglandin D2 ↑ Thromboxane A2 ↑ 20-Hydroxy-leukotriene B4 ↑ Linoleic acid ↑ 9,10-Epoxyoctadecenoic acid ↑ 12,13-EpOME ↑ Paraxanthine ↑ Theobromine ↑	The metabolites were mainly involved in linoleic acid metabolism, arachidonic acid metabolism and caffeine metabolism
		15 healthy individuals			Prostaglandin E2; Prostaglandin H2; Prostaglandin D2; Thromboxane A2; 20-Hydroxy-leukotriene B4; Linoleic acid; 9,10-Epoxyoctadecenoic acid; 12,13-EpOME; Paraxanthine; Theobromine;		
Hearing loss							
Trinh et al.	2019	19 patients affected by sensorineural hearing loss:	Perilyph fluid	LC-MS/MS	N-acetylneuraminate, Glutaric acid, l-cystine, 2-methylpropanoate, Butanoate, Xanthine, l-histidine, S-lactate, 4-hydroxy-l-proline, Serotonin, 2-deoxy-D-Glucose, N-acetyl-l-Alanine; l-proline; Taurine	Patients >12 years: N-acetylneuraminate ↑	
		10 patients ≤12 years					
		9 patients >12 years					
Meniere’s disease							
Di Bernardino F. et al.	2018	26 patients with definite unilateral MD: 14 patients symptomatic and 12 asymptomatic 20 healthy volunteers	Urine	Double sugar test and fecal calprotectin	Lactulose and mannitol absorption	In the symptomatic MD symptomatic: Lactulose and mannitol absorption ↑	
					Fecal calprotectin (FC)	FC ↑	
Obstructive sleep apnea (OSA)							
Xu et al.	2016	60 OSA patients	Urine	UPLC-QTOF-MS coupled with GC-TOF-MS	3-hydroxybutyric acid; 3-methyl-3-hydroxybutyric acid; 4-hydroxypentenoic acid; Lactic acid; Myoinositol; 2-Butenedioic acid	OSA patients compared to SS and control groups:	
		30 simply snored patients (SS)				3-hydroxybutyric acid↑ 3-methyl-3-hydroxybutyric acid ↑ 4-hydroxypentenoic acid ↑	
		30 controls				OSA patients compared to control groups: lactic acid ↑ myoinositol ↑ 2-butenedioic acid ↓	
Lebkuchen et al.	2018	37 patients with OSA	Plasma	MALDI-TOF-MS	Glycerophosphoethanolamines (PE-35:1); Lyso-phosphocholines (LPC-27:1); Sphingomyelin (SM-d18:1/12:0); Glutamic acid, Deoxy sugar; Arachidonic acid; Diacylglycerols (DAG); Glycerophosphocholines (PC); Glycerophosphates (PA)	PE-35:1 ↑ LPC-27:1 ↑	
						SM-d18:1/12:0 ↑	
						Glutamic acid ↑	
		16 controls				Deoxy sugar ↑	
						Arachidonic acid ↑	
						DAG ↓ PC ↓ PA ↓	
Pinilla et al.	2022	206 subjects: 142 OSA subjects [apnea-hypopnea index ≥15 events/hour after polysomnography (PSG)]	Serum	UPLC-QTOF-MS	Glycerophospholipid (cardiolipin (CL); Phosphatidylcholine (PC-P (36:4)); Phosphatidylethanolamine (PE)); Sterols (bile acids); Oxylipids; 25-Cinnamoyl vulgaroside; Glycocholic acid; Bilirubin	CL ↑ PC-P (36:4) ↑ PE ↑ Sterols (bile acids) ↑ Oxylipids ↑	The metabolites were mainly involved in glycerophospholipid metabolism, primary bile acid biosynthesis, linoleic acid metabolism, α-linolenic acid metabolism and glycosylphosphatidylinositol acid metabolism
						Effect of CPAP treatment on the circulating profile of OSA patients:	
						25-Cinnamoyl vulgaroside ↑ Glycocholic acid ↑ Bilirubin ↑	
Xu et al.	2018	30 pediatric subjects with OSA compared to a control group	Urine	UPLC-Q-TOF-MS coupled with GC-TOF-MS	Carbohydrates; Amino acid; Metabolites of microbial origin; Vitamins; Citrulline; Ornithine; 7-Methylguanine; Adenine; Uridine; 3,4,5-Trihydroxypentanoic acid; Quinic acid; Glycerol phosphate; Butanoate; Glucuronic acid; 2-Methylacetoacetic acid; 3-Methyl-2-pentenedioic acid; 3-pyridylacetic acid; Methylcitric acid; Oxalic acid	Increase in biomarker candidates in pediatric OSA patients ↑	The metabolites were involved in amino acid metabolism, carbohydrate metabolism, microbial metabolism, vitamin metabolism, ornithine cycle, nucleic acid metabolism, fatty acid metabolism, butanoate metabolism, and bilirubin metabolism
Noise trauma							
Ji et al.	2019	CBA/J mice (aged 8–12 weeks) subject to exposure to octave band noise (8–16 kHz)	Tissue	UHPLC-QqQ coupled with LC-MS/MS	Nucleotides; Cofactors; Carbohydrates; Glutamate; Amino acids; Cytosine; N-methyl-L-glutamate; L-methionine; L-arginine	Nucleotides ↑ Cofactors ↑ Carbohydrates ↑ Glutamate ↑	The metabolites were involved in metabolism of alanine, aspartate, purine, glutamine and glutamate, and the metabolism of phenylalanine, tyrosine and tryptophan
						Amino acids ↓	
						Change in metabolites with respect to the intensity and duration of noise exposure:	
						Cytosine ↑ N-methyl-L-glutamate ↑	
						L-methionine ↓	
						L-arginine ↓	
Miao et al.	2021	62 NIHL patients	Plasma	UHPLC-Q-TOF MS	Homodeoxycholic acid; Quinolacetic acid; 3,4-dihydroxymandelic acid; PE (15: 0/20: 2 (11Z, 14Z)); PC (15: 0/18: 1 (11Z)); PI (O-20: 0/18: 0)	Homodeoxycholic acid ↑ Quinolacetic acid ↑ 3,4-dihydroxymandelic acid ↑	Autophagy pathway was particularly involved in patients with NIHL
		62 Controls				PE ↓ PC ↓ PI ↓	

### Head and neck cancers

The annual incidence of head and neck cancer (HNC) exceeds 500,000 cases worldwide and accounts for approximately 3% of adult tumors (Johnson et al., 2011). HNC is an epithelial malignancy of the aerodigestive tract and can metastasize to different sites. Approximately 75% of HNCs are oral cancers, and 90% of oral cancers are diagnosed as oral squamous cell carcinomas (OSCC) (Rezende et al., 2010).

Although most risk factors for HNC have been identified, including tobacco, alcohol, and human papillomavirus (HPV) infection (Gillison, 2004; Schmidt et al., 2004), the prognosis for HNC has not improved. This might be because these risk factors alone cannot entirely explain the observed HNC incidence, and some patients are not classified in these risk categories. In addition, other unidentified factors may play essential roles in tumorigenesis and metastasis of HNC (Mao et al., 2004).

Moreover, cancer is considered a metabolic disease marked by cellular metabolism alterations; thus, metabolomics may give insight for understanding the mechanistic origin of altered metabolism. In one of the first studies performed (Almadori et al., 2007) the Authors collected saliva samples from 127 subjects: 50 patients with oral and pharyngeal squamous cell carcinomas and 77 healthy controls. In all the samples the concentration of glutathione was evaluated by HPLC method and was reported to be significantly increased in the oral and pharyngeal SCC group. Oxidative stress has long been implicated in cancer development and progression, suggesting that the increase of glutathione regards its antioxidant action which might counteract the carcinoma growth (Traverso et al., 2013).

Sugimoto et al. (2010) performed capillary electrophoresis time-of-flight mass spectrometry (CETOF-MS) on saliva samples collected from 69 oral cancer patients and 89 healthy controls. Twenty-eight metabolites were significantly different between the two groups (Sugimoto et al., 2010). Salivary polyamine levels were markedly higher among these metabolites in the oral cancer group. Polyamines are organic cations derived from amino acids essential for the growth and development of a range of mammalian tissues and in remodeling processes associated with tissue repair. Their association with cell growth and cancer was first reported in the late 1960s by Russell and Synder (1968) who recorded high levels of ornithine decarboxylase (the first enzyme in the polyamine synthesis pathway) in regenerating rat liver and in several human cancers. Gerner and Meyskens (2004) suggested that quantitative information of the panel of metabolites and their combinations could predict disease susceptibility and may contribute to identify promising biomarkers for medical screening. Tiziani et al. (2009) performed an NMR metabolomics study on blood serum collected from 15 OSCC patients and 10 controls. OSCC patients were characterized by an abnormal metabolic activity with a high rate of glycolysis and increased levels of ketone bodies toward lipolysis. In contrast, a decreased rate of the Krebs cycle suggested the presence of a typical metabolic signature of cancer named the “Warburg effect,” in which tumors rely on glycolysis to provide adenosine triphosphate, nucleotides, lipids, and amino acids for the growth of cancer cells even under aerobic conditions. In addition, more and more studies have recently confirmed that while glycolysis is the primary energy source of cancer cells, it is also involved in activating oncogenes such as phosphatidylinositol 3-kinase and hypoxia-inducible factor-1 alpha (Liu et al., 2021).

In a GC/MS metabolomic analysis of serum and tissue samples obtained from patients affected by squamous cell carcinoma of the head and neck (HNSCC), 109 metabolites were identified. Interestingly, metabolites related with glycolytic pathways (i.e., glucose) were less represented in the tissues (see Warburg effect), whereas amino acids (i.e., valine, tyrosine, serine, and methionine) were expressed at higher levels in the tissues than in the serum (Yonezawa et al., 2013). A tentative explanation regarded the activation of gluconeogenesis, which is the process of glucose synthesis by non-carbohydrate precursors, one of which includes the identified amino acids (Taherizadeh et al., 2020).

### Allergic rhinitis

Allergic rhinitis (AR) is an inflammatory condition of the nose triggered by allergens, such as pollen, dust, mold, or flakes of skin from certain animals. It is characterized by clinical signs such as nasal congestion, clear rhinorrhea, sneezing, postnasal drip, and nasal pruritis. It is a very common condition, estimated to affect around one in six individuals and it is associated with significant morbidity, loss of productivity, and health-care costs. A recent consensus document by the European Academy of Allergy and Clinical Immunology recognized the need to “improve the process of drug development, biomarkers and diagnostics for allergic diseases and asthma” (Agache et al., 2019).

The application of metabolomics strategies to discover new biomarkers in AR is suggested for both the characterization of naive patients and monitoring patients under different stages of therapy. In particular, concerning metabolomics studies, AR naive patients are a rare, unique group that, once evaluated, can indicate the metabolites altered by the disease. However, AR patients are progressively treated with different drugs aiming to achieve disease control, which in turn may alter systemic signatures associated with disease progression.

One example is the study performed by Ma et al. (2020) who analyzed the serum metabolomics profile of 28 AR patients compared with 28 healthy controls (twenty-six patients received drug treatment before samples collection). Samples were examined using an ultra-high performance liquid chromatography-quadrupole time-of-flight MS (UHPLC/Q-TOF-MS), and the acquired spectra were then analyzed by a multivariate statistical analysis approach that recognized ten metabolites significantly altered in AR patients. Bilirubin, hypoxanthine, 15(S)-hydroxyeicosatetraenoic acid [15(S)-HETE], hexadecenoic acid, and urate were more represented, whereas 13(S)- hydroperoxyoctadecadienoic acid [13(S)-HPODE], leukotriene D4, Lstercobilinogen, N-succinyl-L-diaminopimelic acid, and chlorophyll were downregulated. Pathway analysis showed that these changes in metabolites mainly involved three pathways, namely, porphyrin and chlorophyll metabolism, arachidonic acid metabolism (AA), and purine metabolism. Author conclusions indicated that although drug therapy is known to block different pathways of AR patients effectively, the identified pathways, such as the AA metabolism is a metabolic pathway associated with an inflammatory reaction. Therefore, further studies may need to verify the value of the identified potential biomarkers of AR.

Zheng et al. (2021) focused on the drug treatment of 73 AR patients who were simultaneously sensitized by Der p and Der f with single-mite subcutaneous immunotherapy (SM-SCIT) or double-mite subcutaneous immunotherapy (DM-SCIT). Both treatments with SM-SCIT (35 patients) and DM-SCIT (38 patients) consisted of three administrations, leading to 219 serum samples. All the serum samples were analyzed using ultra-high-performance liquid chromatography-quadrupole-time of flight mass spectrometry (UHPLC-Q-TOF/MS), resulting in 57 metabolites identified and relatively quantified. Among them, 31 metabolites were found significantly decreased from T0 to T1 and T2. Interestingly, 15(S)- hydroxyeicosatetraenoic acid (HETE), 5(S)-HETE, 12(S)- hydroxyeicosapentaenoic acid (HEPE), and 13- hydroxyoctadecadienoicacid (HODE) were significantly lower after treatment, both in SM-SCIT and DM-SCIT patients, whereas 11(S)-HETE, 8(S)-HETE, 5(S)-HEPE, AA, and eicosapentaenoic acid (EPA) decreased in DM-SCIT patients, and only 9(S)- hydroperoxylinoleic acid (HPODE) increased in SM-SCIT patients. Further analysis revealed that five metabolites [5(S)-HETE, 8(S)-HETE, 11(S)-HETE, 15(S)-HETE, and 11-hydro TXB2] showed significant differences after approximately 1 year of treatment in SM-SCIT or DM-SCIT, and these metabolic changes were correlated with the magnitude of rhino-conjunctivitis and quality of life questionnaire improvement, respectively. In conclusion, the authors suggested that both SM-SCIT and DM-SCIT treatments had therapeutic effects on patients with AR causing significant metabolic changes, and that metabolomics could help monitor these changes.

An intriguing study was performed by Yuan et al. (2022) who aimed at gaining insight into the role of microflora and the metabolic characteristics of AR patients in order to identify new diagnostic biomarkers. In this study, the microorganisms of the nasal airways and the serum collected from 28 patients and 15 healthy individuals were explored using both 16S rRNA sequencing and UPLC-Q/TOF-MS/MS untargeted metabolomics. The AR group showed a significantly higher abundance of 1 phylum (Actinobacteria) and seven genera than healthy controls (Klebsiella, Prevotella, and Staphylococcus were significantly increased in AR, while in HC the most prevalent genera were Moraxella, Haemophilus, Streptococcus, and Flavobacterium). This finding aligns with the current literature where AR cohorts present a distinct gut microbiome profile, marked by a reduced microbial diversity and altered abundance of specific gut microbes compared to controls. The existence of unique microbiome patterns may help identifying the microbiota-host relationship, improving the knowledge of how the microbiome regulates immune homeostasis. This may guide the development of potential therapeutic options for treating allergies, such as dietary intervention, probiotics administration, and fecal transplant. Regarding the metabolomics analysis of the serum, the authors found 26 different metabolites and 16 perturbed metabolic pathways. Among the latter, the three most significantly altered pathways were linoleic acid metabolism, arachidonic acid metabolism, and caffeine metabolism. Authors suggested that these coordinated approaches of microbiome and serum metabolomics resulted in highly correlated data and could be used as biomarkers for the diagnosis of AR.

### Obstructive Sleep Apnea

Obstructive sleep apnea (OSA) is the most common sleep breathing disorder. Individuals with OSA show obstruction of the upper airways during sleep, which reduces intrathoracic pressure intermittent hypoxia, and consequently sleep quality is negatively affected (Dempsey et al., 2010).

OSA is prevalent in males and obese subjects, leading to a greater risk of cardiovascular, metabolic (insulin resistance), and cognitive disorders (Sjöström et al., 2002; Yaffe et al., 2011).

To date, the gold standard for the diagnosis of OSA is nocturnal polysomnography (PSG). However, the PGS is a time-consuming and relatively expensive diagnostic technique, and its use for the prevention, early diagnosis, and treatment of OSA is therefore limited (Semelka et al., 2016). In recent years, several studies have been published to search for new biomarkers to better understand the patho-physiological mechanisms underlying OSA and develop preventive strategies and therapeutic interventions. Xu et al. (2016) conducted a study by combining ultra-performance liquid chromatography and gas chromatography coupled with quadrupole time-of-flight mass spectrometry to evaluate the metabolomic profile of urine samples from three groups: 1) OSA patients 2) a control group (age and weight-matched), and 3) patients who snore at night. All the subjects recruited into the study underwent PSG at night and were classified into the three different clusters mentioned above (Xu et al., 2016).

Urinary metabolomics analysis showed several fatty acids, such as 3-hydroxybutyric acid, 3-methyl-3-hydroxybutyric acid, and 4-hydroxypentenoic acid elevated in OSA patients compared to snoring patients and the control group. A tentative explanation about the increase in fatty acids could be due to the marked lipolysis caused by intermittent hypoxemia in OSA patients, which can compromise the anti-lipolytic function of insulin, resulting in important lipolysis. Furthermore, biochemical blood measurements showed that patients with OSA had dyslipidaemia as evidenced by higher levels of total cholesterol (TC), triglycerides (TG), low-density lipoprotein cholesterol (LDL-C), and lower levels of high-density lipoprotein cholesterol (HDL). This could partially explain the eventual development of cardiovascular and metabolic diseases in patients with OSA. The urinary metabolomic analysis also indicated an increase in lactic acid and myoinositol and a decrease in 2-butenedioic acid, an intermediate of the TCA cycle. Due to hypoxia, TCA may not provide sufficient ATP in OSA patients and therefore glycolysis increases its activity to compensate. Furthermore, intermittent hypoxia leads to excessive oxidative stress and nuclear and mitochondrial dysfunction. Thus, the increase in myoinositol may be due to the compensatory response triggered by skeletal muscle injury in patients with intermittent hypoxia induced OSA (Nanduri et al., 2015).

Lebkuchen et al. (2018) conducted a plasma metabolomics study of 37 patients with OSA versus 16 healthy controls. The lipidomic profile was analysed with the MALDI-TOF-MS technique, while the same samples aqueous fractions were analysed using a targeted LC-MS analysis. The results indicated that patients with OSA were characterized by higher levels of glycerophosphoethanolamines (PE-35:1), lyso-phosphocholines (LPC-27:1), sphingomyelin (SMd18:1/12:0), glutamic acid, deoxy sugar and arachidonic acid, and lower levels of diacylglycerols (DAG), glycerophosphocholines (PC), and glycerophosphates (PA). A tentative interpretation concerning the increase of arachidonic acid may be due to the activation of phospholipase A2 that can act as a substrate for cyclooxygenase enzymes in the generation of prostaglandin E2. In particular, prostaglandin E2 seemed to regulate vascular resistance, myocardial ischemia, myocarditis, and other cardiovascular pathologies (Hochachka, 1986; Suzuki et al., 2011).

Furthermore, the authors reported a decrease in glutamate, which was highlighted by several studies regarding how arachidonic acid could produce a slow-developing inhibition of glutamate absorption. In addition, the alteration of sphingomyelin (SM d18: 1/12: 0) may be due to hypoxia’s severity, which determines the activation of sphingomyelin phosphodiesterase (SMase) with its consequent increase in plasma concentration in patients with OSA (Barbour et al., 1989).

A recent study (Pinilla et al., 2022) included 206 subjects, 142 OSA patients, who underwent plasma samples collection and metabolomic and lipidomic analysis by using ultra-high performance liquid chromatography coupled to electrospray ionization quadrupole time of flight tandem mass spectrometry. In addition, in a small group of one hundred patients, the impact of continuous positive airway pressure (CPAP) treatment for 6 months was evaluated. The results indicated that the most affected lipid classes in OSA patients were glycerophospholipids cardiolipin (CL), phosphatidylcholine PC-P (36:4), phosphatidylethanolamine (PE), sterols (bile acids), and fatty acid derivatives (oxylipids), which belonged to various pathways including glycerophospholipid metabolism, primary bile acid biosynthesis, linoleic acid metabolism, α-linolenic acid metabolism and glycosylphosphatidylinositol acid metabolism.

By acting as a scavenger of ROS, the authors interpreted the increase in PC-P (36:4) as a marker to indicate the reduction of oxidative stress in OSA patients. At the same time, the role of CL and chenodeoxycholic acid, a major component of bile acids, may be intended as an adaptive mechanism to counteract the damage deriving from hypoxia. CL is a glycerophospholipid that plays a crucial role in the integrity and activity of the mitochondrial electron transport chain complex and, consequently, in cellular bioenergetic processes. At the same time, chenodeoxycholic acid acts as a signalling molecule, regulating the factor Hypoxic inducible -1-alpha (HIF-1-alpha) in hypoxic conditions (Paradies et al., 2014; Moon et al., 2015). Subsequently, the authors explored the effect of CPAP treatment on plasma samples collected from one hundred OSA patients, which resulted also altered for 25-Cinnamoyl-vulgaroside, Glycocholic acid, Bilirubin, and two unknown features. The authors suggested that persistent alteration of some metabolites after CPAP therapy may be due to the inability of CPAP to fully reverse all OSA-induced alterations (Marcus et al., 2012).

Xu et al. (2018) studied the metabolomics profile of 30 paediatric subjects affected by OSA compared to a control group. Urine samples were collected and analysed with UPLC-Q-TOF-MS and GC-TOF-MS resulting in 57 discriminant metabolites belonging to the following pathways: amino acid metabolism, carbohydrate metabolism, microbial metabolism, vitamin metabolism, ornithine cycle, nucleic acid metabolism, fatty acid metabolism, butanoate metabolism, and bilirubin metabolism (Xu et al., 2018). In addition, a 16S rRNA metagenomic analysis of the oral cavity was conducted on the same patients, which highlighted five predominant phyla in OSA patients: Firmicutes, Proteobacteria, Bacteroidetes, Fusobacteria, and Actinobacteria, which constituted 98.6% of the total oral microbiota. The authors identified that the alteration of the urinary profile was strongly correlated with the altered microbiome and hypothesized that changes in the oral microbiome may lead or exacerbate OSA-related metabolic disorders.

### Hearing loss

Hearing loss (HL) is an etiologically heterogeneous disorder that affects around 12% of the world’s population, above all older subjects. HL is categorized as sensorineural, conductive, and mixed. There are many causes of HL: in the paediatric population, otitis media is the most frequent cause but 1-3 out of 1,000 patients suffer from sensorineural HL; in the adult and elderly population the principal cause of HL is age (presbycusis) followed by acoustic trauma. In many cases the aetiology remains uncertain with a possible genetic origin. However, phenotypic investigations are needed to better understand the true cause of HL.

In this regard, a study by Carta et al. (2017) using a 1H-NMR approach evaluated the urinary metabolome of 26 subjects: 21 with idiopathic sudden sensorineural HL and five healthy controls. Among the assessed patients, patients were sub-classified as those who did not recover from hearing loss after steroids and those who recovered after treatment. Urinary metabolome significantly differed between responders and non-responders, with B-Alanine, 3-hydroxybutyrate, and TMAO more represented in the former group, while citrate and creatinine were less represented. Authors suggested that the metabolomics characterization of idiopathic sudden sensorineural hearing loss may identify a predisposed effective action of steroids allowing the recovery after treatment (Carta et al., 2017).

A different biofluid was used by Trinh et al. (2019) who performed the metabolomics characterization on the perilymph fluid of 19 patients affected by sensorineural HL. The perilymph fluid was collected during cochlear implantation surgery analysis and was successively analysed by Liquid Chromatography-High-Resolution Mass spectrometry, which identified 106 different metabolites. A supervised statistical classification based on HL patients’ belonging to groups with age ≤12 and >12 years identified several discriminant compounds, such as: N-acetylneuraminate, glutaric acid, cystine, 2-methylpropanoate, butanoate, and xanthine. The authors indicated that the age of patients was one of the main metabolic discriminant parameters, which was characterized by alteration in energetic metabolism, increased inflammation, and oxidative stress. In addition, an interesting correlation resulted between the metabolite n-acetylneuraminate and the duration of hearing loss. According to the authors, these findings may uncover the pathophysiological pathways associated with sensorineural HL.

One of the most frequent causes of HL is acoustic trauma. Approximately 180 million people worldwide live with HL due to daily exposure to potentially harmful factors, including noise trauma. The effect of noise on the auditory system (hypoacusis) is directly related to the sound level and the duration of exposure so that, once certain limits are exceeded, there is a risk of irreversible damage to the hearing system (Albera et al., 2010). To date, despite the critical impact of noise-induced HL, the molecular mechanisms underlying the damage to the inner ear due to noise trauma are still not fully understood. Therefore, the application of novel tools such as metabolomics might help the identification of potential key molecular pathways related to noise-induced HL.

Ji et al. (2019) studied the metabolomics profile of temporal bone of CBA/J mice (aged 8–12 weeks) with normal hearing, subjected to exposure to octave band noise (8–16 kHz). The mice were divided into several groups, the noise levels ranged from 98 to 110 dB SPL, and the duration of the noise ranged from 1 h to 2 h. The metabolomics profile of the mice exposed to the noise source was compared with a control group that received no noise exposure. Tissue samples were analysed with UHPLC and Triple Quadrupole (QqQ) Mass Spectrometer (LC-MS/MS), resulting in 40 discriminant metabolites between the control and the noise-exposed groups. Notably, 25 metabolites including nucleotides, cofactors, carbohydrates, and glutamate were upregulated, while 15 metabolites, mainly amino acids, were downregulated in mice exposed to noise stimulation. The metabolic pathways analysis showed that the metabolism of alanine, aspartate, purine, glutamine and glutamate, and the metabolism of phenylalanine, tyrosine and tryptophan were the most involved. In addition, the authors investigated the changes in metabolites concerning the intensity and duration of noise exposure. The results showed that a noise intensity of 98–110 dB caused an alteration in the concentration of cytosine, N-methyl-L-glutamate, L-methionine and L-arginine. The results were validated with SPL noise of 100 dB for 2 h that resulted in 39 altered metabolites, including glutamate, methionine, arginine, tryptophan, xanthurenic acid NAD + and oxidized glutathione (SSG). Regarding the altered metabolic profile, an explanation to the pathogenesis of hidden HL involves the metabolite glutamate, the major afferent neurotransmitter in the auditory system. In fact its excessive release could activate receptors in the cochlea, causing excitotoxicity and damage to the afferent terminals of the hair cells. Furthermore, the noise-exposed mice showed downregulation of xanthurenic acid, an inhibitor of the vesicular glutamate transporter VGLUT24, suggesting that VGLUT activity may increase after the noise (Hakuba et al., 2000). Moreover, the presence of methionine and antioxidant systems such as NAD + and FAD + may be linked to the glutathione pathway, which may counteract the noise-induced cochlear damage (Campbell et al., 2007; Neale et al., 2013).

Recently Miao et al. (2021) analysed the plasma samples of 62 NIHL patients, and 62 normal-hearing controls using ultrahigh-performance liquid chromatography coupled with quadrupole time-of-flight tandem mass spectrometry (UHPLC-Q-TOF MS). The multivariate statistical analysis allowed to discriminate between the two groups of patients and identify the metabolites responsible for the separation. Among them, homodeoxycholic acid, quinolacetic acid and 3,4-dihydroxymandelic acid were more represented in patients with noise-induced HL, while lipids, such as phosphatidylethanolamine (PE), phosphatidylcholine (PC), and phosphatidylinositol (PI) were less represented. Successively, pathways analysis was performed and identified those most involved in the development of NIHL: autophagy, glycerophospholipid metabolism, glycosylphosphatidylinositolanchor biosynthesis, choline metabolism, alpha-linolenic acid metabolism, linoleic acid metabolism, and retrograde endocannabinoid pathway. Therefore, the authors hypothesized that the reduction of PE, PC, and PI, essential antioxidants involved in a variety of physiological functions of the cells, may be due to the excessive production of ROS during noise exposure. Consequently, those metabolites were found to be reduced due to their contribution to the antioxidant defence system. Moreover, the reduced plasma concentration of PE and PC seemed to be correlated with the autophagy pathway. A further clue for this hypothesis was the transcriptomics analysis of three autophagy related genes: PI3K, AKT, and ATG5, less expressed among NIHL patients than among controls. A tentative explanation about the inhibition of PI3K and AKT signalling was linked to the activation of apoptotic signals, which could in turn cause the death of the hair cells in the inner ear and the onset of NIHL. Regarding ATG5, Fujimoto et al. (2017) showed that ATG5 deletion could cause hair cell degeneration and severe congenital hearing loss. Indeed, the level of ATG5 expression was significantly reduced in patients with NIHL compared to controls, suggesting that autophagy could be considered an essential signalling pathway in the development of NIHL (Fujimoto et al., 2017).

### Meniere’s disease

Meniere’s disease (MD) is a chronic inner ear syndrome characterized by cochlear and vestibular dysfunction. The symptoms include sensorineural HL, vertigo, tinnitus, and aural fullness. Vertigo attacks can persist from a few minutes to many hours and can recur at irregular intervals. The prevalence of MD has been estimated between 17 and 513 out of 100,000 individuals depending on gender and geographical localization (Berlinger, 2011). Women are more susceptible than men, and Caucasians are more affected compared with Asians and Africans. These findings suggest the presence of both gender and genetic familial clustering based on ethnic differences. The diagnosis is based on the association of vertigo attacks and the demonstration of sensorineural HL. Today, MD is considered a complex disease, whose pathophysiological mechanisms and optimal therapeutic options have yet to be clarified. In this regard, the application of new strategies for the study of changes in metabolism may help to elucidate MD’s pathophysiology (Albera et al., 2016).

In 2019 Albernaz (2019) collected 98 MD patients evaluating familial history of diabetes, glucose tolerance and insulin titration. The results indicated an alteration of the carbohydrate metabolism, which was hypothesized as a possible aetiology for MD.

Another intriguing clue was recently provided by Di Bernardino et al. (2018) who evaluate the existence of a connection between MD and gut permeability. The rationale was that MD patients may experience non-specific gastrointestinal symptoms associated with otological manifestations. Authors collected twenty-six patients with definite unilateral MD (14 patients were symptomatic while 12 asymptomatic) and 20 healthy volunteers as “control group.” The aim was to test the urinary levels of lactulose and mannitol (double sugar test) and the fecal calprotectin (FC), both markers of altered intestinal permeability, in subjects with definite MD in an active and inactive stage. Interestingly, lactulose and mannitol absorption were significantly increased in the symptomatic MD patients compared to the asymptomatic group and to the controls. FC were also significantly more represented than normal only in the symptomatic group. In conclusion authors suggested that in a certain number of symptomatic MD patients is identifiable the presence of dysbiosis of the gut microbiome which in turn may influences the characteristic vestibular symptoms (Di Bernardino et al., 2018).

## Comments and conclusion

Our manuscript aims at summarizing the most recent evidence in the characterization of the ENT metabolome, allowing for the evaluation of its influences on the metabolism of several diseases. The detection of a panel of biomarkers, each characteristic of a group of ENT diseases (Table 1), proved to be highly promising for early diagnosis, prognosis, and response to therapy.

As an example, regarding head and neck cancers, the authors Sugimoto et al. (2010), Tiziani et al. (2009), and Taherizadeh et al. (2020) even in different specimens (saliva, serum, and tissue) and with different methods, found common alterations in metabolism as highlighted by variations in alanine, betaine, phenylalanine, tyrosine, valine, and choline (Sugimoto et al., 2010), glutamic acid, aspartic acid and serine (Tiziani et al., 2009). As mentioned previously, the biological meaning of most of the shared metabolites concerns the growth and remodelling of cancer tissues.

Concerning allergic rhinitis, in different studies (Zheng et al., 2021; Yuan et al., 2022), the authors using the same approach (UPLC-QTOF-MS), found different metabolites, mostly belonging to the same pathway. However, most metabolites were related to the arachidonic acid metabolism which is considered helpful at mirroring a reduction of inflammation after therapy. Moreover, no shared metabolites were found when considering diseases such as OSA and noise trauma.

It can be stated that generally ENT diseases induce alterations at the level of cellular metabolism, which metabolomics studies can identify. Regrettably, even considering the same disorder, metabolic alterations were highly heterogeneous. For example, the most significant metabolites identified differed between Tiziani et al. (2009), Sugimoto et al. (2010), Zheng et al. (2021), and Yuan et al. (2022). Therefore, the application of metabolomics to develop a tailored medicine, also called precision medicine, although considered crucial for identifying the mechanistic origin of altered metabolism, seems not yet ready for comparing and performing epidemiological studies.

Nevertheless, metabolomics has been extensively studied in several disciplines, as evidenced by more than 50.000 related papers on PubMed until now (PubMed, 2022). Concerns in the research community are still represented by standardization and harmonization of the studies. Today, metabolomics is still considered an emerging technology that is supposed to be increasingly incorporated into the clinical laboratory. However, a bottleneck to advancing the field is the complexity and lack of standard protocols or best practices for metabolomics experiments.

Therefore, it is mandatory to recognize the urgent need to perform comparable, reliable, high-quality and precise quantitative data for targeted or untargeted metabolomics. Identifying metabolic and microbial alterations is considered the new Rosetta Stone of medicine, an epigenetic determinant of human health status, which may help to unlock the obscure signs of several diseases (Ochoa-Reparaz et al., 2009; Noto et al., 2014; Friedland, 2015).

Considering this review article, other limitations for the application of metabolomics for ENT should be considered:

1) most of the studies do not mention the pre-analytical part of a study, such as: how samples were collected, preserved (−20°C or −80°C), and prepared (whether a bacteriostatic agent was added primarily for urine and saliva);
2) analytical protocols should be standardized and recognized by the scientific societies both for the application of metabolomics in the clinical setting and for the possibility to make results comparable;
3) most of the studies are limited to the discovery phase, which means that the number of subjects included are few (usually less than 30), which means that the biological information obtained is preliminary and should be confirmed by further studies, such as validation studies performed on larger cohorts of patients (around 100 subjects).

All the steps mentioned are essential to harmonize the data and make them comparable (Table 2). However, harmonization of the data usually requires substantial effort due to heterogeneity in phenotype definitions. Indeed, metabolomics has a unique data structure that depends on the platform (targeted or untargeted approaches), which determines the analytical strategy. Challenges include pre-processing (alignment, filtering), metabolite identification/annotation, data preparation (centering, scaling, transformation), imputation, and statistical approaches. In conclusion, there is a clear need to establish benchmarks about the pre-analytical and post-analytical steps, and on how the analytic choice is crucial for the type of the study (targeted or untargeted).

**TABLE 2:** A brief summary of the harmonization steps.

Data preprocessing	Data analysis	Results
• Quality control	• Discriminant analysis	• Metabolite annotation
• Metabolite centering and scaling	• Correlation analysis	• Minimum reporting requirements
• Metabolite transformation	• Pathway analysis	• Meta-data
	• Calculation of biomarker performance	
	• Multiple testing correction	
	• Validation of results	

### Future research directions

Concerning future research directions, we are encouraged by the increased number of ENT studies published recently using -omics approaches such as metabolomics, which may lead to significant improvements in the prediction accuracy of new biomarkers. As a result, these observations could allow scientists to identify shared protocols (for example, depending on the biofluids used) which will lead to univocal interpretation for a better understanding of the pathophysiological mechanisms underlying ENT diseases.
